# Intraoperative Heparin Dose Adjustment in Chronic Limb-Threatening Ischemia Associated with Antiphospholipid Syndrome: A Case Report

**DOI:** 10.3400/avd.cr.26-00090

**Published:** 2026-08-28

**Authors:** Toru Kikuchi, Yohei Yamamoto, Natsuho Maekawa, Kazuki Tsukuda, Ai Kazama, Yoshiki Wada, Tsuyoshi Ichinose, Toshifumi Kudo

**Affiliations:** 1 Division of Vascular Surgery, Department of Cardiovascular Surgery, Institute of Science Tokyo, Tokyo, Japan; 2 Division of Vascular Surgery, Nissan Tamagawa Hospital, Tokyo, Japan

**Keywords:** antiphospholipid syndrome, chronic limb-threatening ischemia, activated clotting time

## Abstract

Antiphospholipid syndrome (APS) is an autoimmune prothrombotic disorder characterized by recurrent arterial and venous thrombosis, as well as pregnancy complications. Laboratory findings often show prolongation of activated partial thromboplastin time, which complicates monitoring during heparin therapy. We present the case of a 69-year-old woman with APS who developed chronic limb-threatening ischemia. She underwent successful bilateral lower-extremity open surgical revascularizations guided by a preoperatively obtained heparin–activated clotting time (ACT) titration curve to define individualized intraoperative anticoagulation targets. This method offered a straightforward and dependable strategy for personalized anticoagulation management and may enhance the safety of peripheral arterial revascularization in patients with APS.

## Introduction

Antiphospholipid syndrome (APS) is an autoimmune prothrombotic disorder characterized by recurrent arterial and venous thrombosis, as well as pregnancy complications. Laboratory findings commonly reveal prolongation of activated partial thromboplastin time (APTT), which complicates the monitoring of heparin therapy. Intraoperative activated clotting time (ACT) may also be unpredictably prolonged, rendering standard heparin dosing unreliable. We present a case in which a preoperative heparin–ACT titration curve was used to establish individualized intraoperative anticoagulation targets during lower-extremity open surgical revascularization in a patient with APS.

## Case Report

A 69-year-old woman presented with progressive gangrene of the right toes and rest pain in the left lower extremity. The patient had no significant past medical history. Contrast-enhanced computed tomography (CT) revealed occlusion of the right common femoral artery (CFA) and an occlusion extending from the left common iliac artery (CIA) to the external iliac artery (EIA), along with bilateral superficial femoral artery (SFA) occlusions. Although no clinical signs of bleeding were observed, laboratory testing revealed a markedly prolonged APTT of 111.1 s. Further evaluation of the prolonged APTT demonstrated an inhibitor pattern on the mixing study. Although the anti-cardiolipin IgG antibody level was within the reference range (6.6 U/mL; reference range, <12.3 U/mL), the anti–β2-glycoprotein I antibody level was elevated (10.4 U/mL; reference range, <3.5 U/mL) and the lupus anticoagulant (LA) ratio (2.5; reference range, <1.3) was positive. These findings raised suspicion of APS. The patient was scheduled for staged surgical revascularization for chronic limb-threatening ischemia (CLTI).

The initial procedure consisted of right CFA endarterectomy and a right CFA–above-knee popliteal artery bypass using a 6-mm PROPATEN graft (W. L. Gore & Associates, Flagstaff, AZ, USA).

**Fig. 1:**
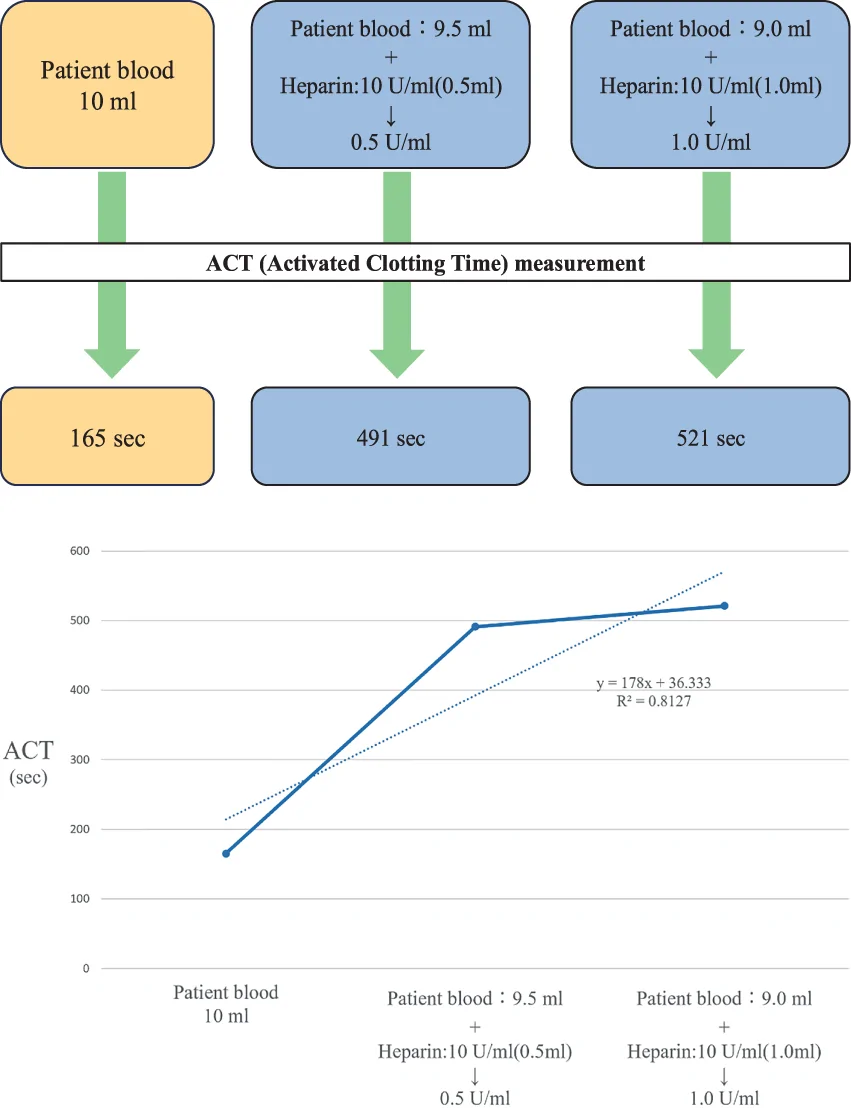
Change in ACT in response to heparin dosage and the heparin–ACT titration curve during the first surgery. ACT, activated clotting time

Intraoperative anticoagulation was guided using a patient-specific heparin–ACT titration curve. Preoperatively, 3 ACT measurements were obtained using the ACT Plus analyzer (Medtronic, Minneapolis, MN, USA): (1) native blood, (2) blood adjusted to a heparin concentration of 0.5 U/mL, and (3) blood adjusted to a heparin concentration of 1.0 U/mL. The corresponding ACT values were (1) 165, (2) 491, and (3) 521 s, respectively. Based on these results (**Fig. 1**), an initial intravenous bolus of 2500 U (50 U/kg) of heparin was administered, targeting an intraoperative ACT exceeding 500 s. The ACT after the initial heparin administration was 532 s. ACT was measured repeatedly as needed, and additional heparin was administered to maintain the target ACT. At 1 hour after the initial heparin administration, the ACT had decreased to 481 s; therefore, an additional 1000 U (20 U/kg) of heparin was administered, resulting in a cumulative heparin dose of 3500 U (70 U/kg).

When serological testing was repeated 12 weeks later, the anti-cardiolipin IgG antibody level remained within the reference range (9.5 U/mL), whereas the anti–β2-glycoprotein I antibody level remained elevated (9.2 U/mL), and the LA ratio remained positive (3.1), confirming persistent positivity. Based on the 2023 American College of Rheumatology/European Alliance of Associations for Rheumatology (ACR/EULAR) classification criteria, the patient was diagnosed with double-positive APS.^1,2)^

**Fig. 2:**
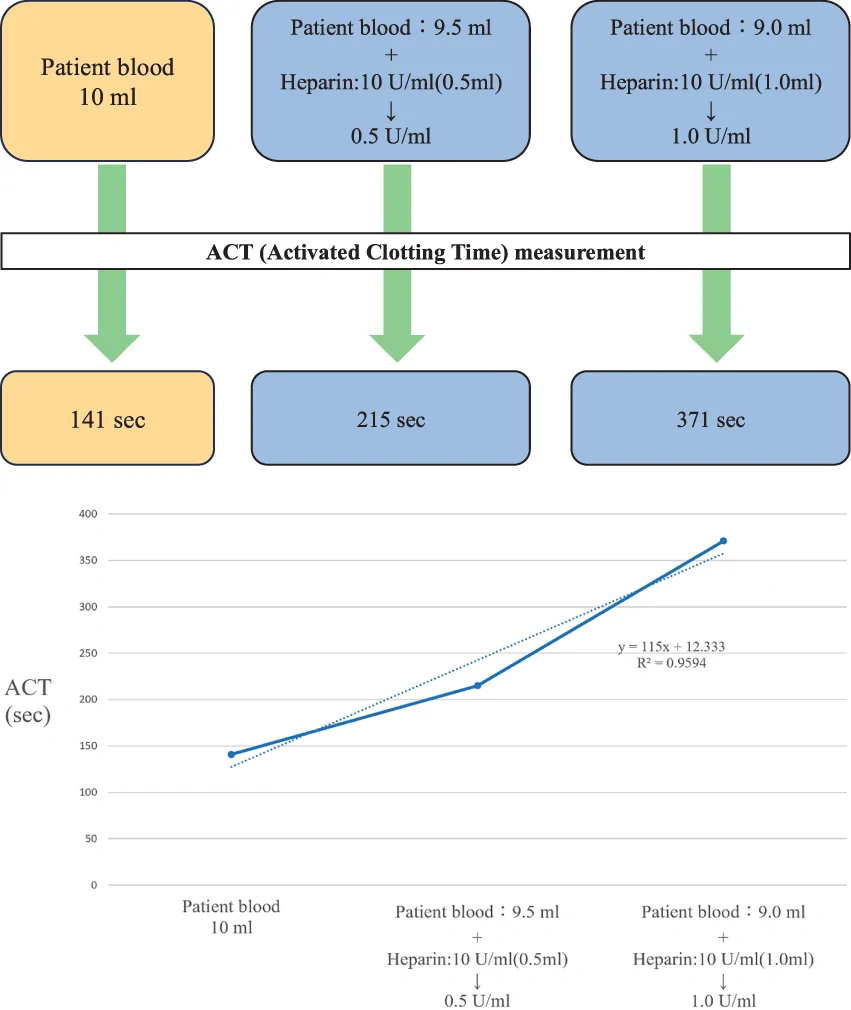
Change in ACT in response to heparin dosage and the heparin–ACT titration curve during the second surgery. ACT, activated clotting time

The patient subsequently underwent left lower-extremity revascularization, including a right EIA–left CFA bypass using an 8-mm PROPATEN graft and a left CFA–above-knee popliteal artery bypass using a 6-mm PROPATEN graft. Preoperative ACT measurements were (1) 141, (2) 215, and (3) 371 s (**Fig. 2**). Based on these findings, an initial heparin dose of 2500 U (50 U/kg) was administered, targeting an ACT exceeding 200 s. The ACT after the initial heparin dose was 215 s, and additional heparin was administered to maintain the target range. At 1.5 hrs after the initial dose, the ACT was 182 s; therefore, an additional 1000 U (20 U/kg) of heparin was administered. The cumulative heparin dose was 3500 U (70 U/kg). Both procedures were completed without complications.

**Fig. 3:**
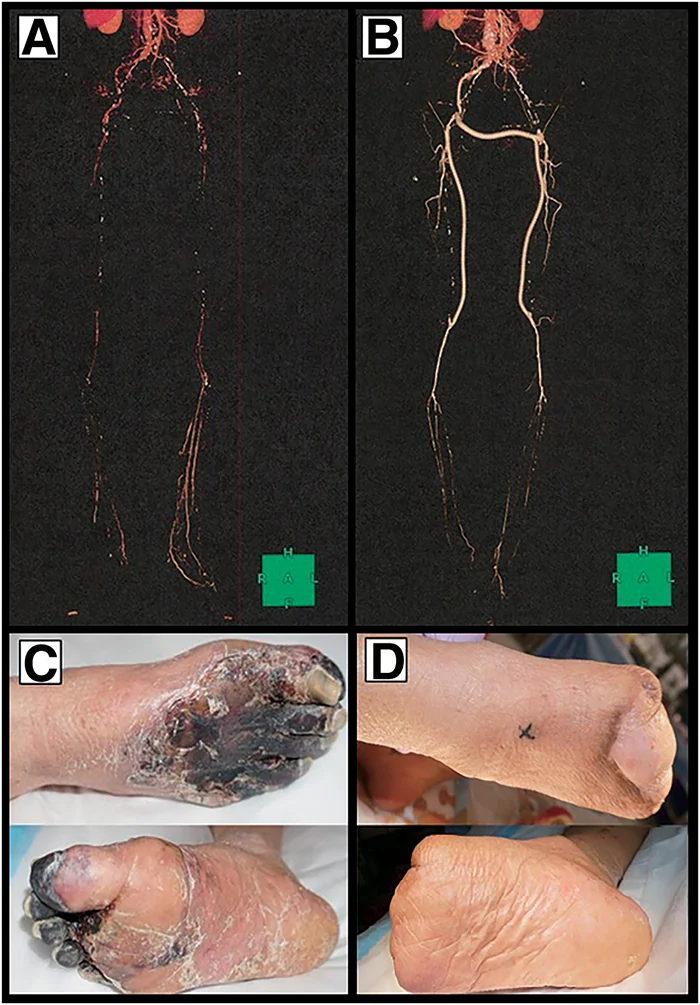
Preoperative and postoperative contrast-enhanced CT and foot findings. (**A**) Three-dimensional reconstructed images from preoperative contrast-enhanced CT. (**B**) Three-dimensional reconstructed images from postoperative contrast-enhanced CT. (**C**) Preoperative photograph of the right foot. (**D**) Photograph of the right foot, which had fully healed following revascularization surgery and metatarsectomy. CT, computed tomography

The patient later underwent a right foot transmetatarsal amputation for persistent gangrene, which healed without adverse events. Preoperative contrast-enhanced CT imaging (**Fig. 3A**) and postoperative contrast-enhanced CT imaging (**Fig. 3B**) confirmed that revascularization was successful. Clinical photographs showed black necrosis of the right foot before surgery (**Fig. 3C**), which subsequently healed completely following revascularization and metatarsectomy (**Fig. 3D**). Postoperatively, antithrombotic therapy consisted of aspirin in combination with warfarin, with a target international normalized ratio of 2.0–3.0. The patient remains ambulatory and clinically stable 12 months after surgery.

## Discussion

APS is an autoimmune-mediated acquired thrombophilia characterized by the presence of antiphospholipid antibodies and clinical manifestations that include arterial and venous thrombosis, pregnancy-related complications, thrombotic microangiopathy, and thrombocytopenia. It is particularly important to perform appropriate diagnostic testing to establish a differential diagnosis in patients with a history of autoimmune diseases, such as systemic lupus erythematosus (SLE), or those presenting with clinical manifestations, including arterial or venous thrombosis.^3,4)^ Diagnosis requires fulfillment of both clinical and laboratory criteria, including the persistent detection of antiphospholipid antibodies such as LA and anticardiolipin antibodies.^5)^ LA interferes with phospholipid-dependent coagulation assays, including APTT, by inhibiting coagulation factors V and X, thereby complicating heparin monitoring.^6)^ The patient fulfilled the 2023 ACR/EULAR classification criteria for APS,^1,2)^ and had no concomitant autoimmune diseases; therefore, she was diagnosed with primary APS.

Anticoagulation management in patients with APS is challenging because commonly used assays such as APTT and ACT may be unreliable. Several strategies have been proposed to guide intraoperative heparin dosing in patients with APS undergoing cardiac surgery, including doubling the baseline ACT,^7)^ protamine titration,^8)^ measurement of anti-factor Xa activity, or construction of a preoperative heparin–ACT titration curve.^9,10)^

Doubling the baseline ACT is a simple and practical approach; however, its accuracy is limited. Protamine titration provides a reliable estimation of heparin requirements but requires specialized equipment, such as the Hepcon-HMS system (Medtronic).^8)^ Anti-factor Xa assays allow precise assessment of circulating heparin levels but are time-consuming and impractical for real-time intraoperative use. Furthermore, when baseline ACT values are already prolonged, doubling the baseline may exceed the upper measurement limit of the assay.

In contrast, constructing a heparin–ACT titration curve requires accurate mixing of blood and heparin, does not rely on specialized equipment, and can be performed with relative ease. Although multiple methods for determining heparin dosing have been reported in the context of cardiac surgery, we found no reports specifically addressing peripheral arterial revascularization.

In this case, intraoperative heparin dosing was individualized by establishing patient-specific target ACT levels using preoperative heparin–ACT titration curves, as described by Seki et al.^9)^ Blood samples adjusted to heparin concentrations of 0.5 and 1.0 U/mL were used because the standard intraoperative bolus of 50 U/kg of heparin administered during arterial clamping corresponds to an estimated circulating heparin concentration of approximately 0.7 U/mL. The target ACT during the first operation was determined not by a predefined threshold but by the preoperatively generated heparin–ACT titration curve. Before the first operation, the preoperative ACT values at heparin concentrations of 0.5 and 1.0 U/mL were 491 and 521 s, respectively. These findings indicated that the standard heparin dose of 50 U/kg, corresponding to an estimated blood heparin concentration of approximately 0.7 U/mL, would achieve an ACT exceeding 500 s. Accordingly, an individualized target ACT exceeding 500 s was selected to ensure adequate anticoagulation. Using the same preoperative heparin–ACT titration, the target ACT for the second operation was set at 200 s.

During cardiopulmonary bypass (CPB), ACT may be prolonged due to hemodilution and hypothermia, potentially resulting in inadequate anticoagulation when ACT is used as the sole indicator. In peripheral arterial revascularization, however, this limitation is less relevant because CPB is not employed.

ACT is a phospholipid-dependent assay that is influenced not only by LA antibodies but also by patient-related factors, including body temperature, platelet count, and coagulation status, as well as by the reagents and equipment used for the assay.^6)^ In this case, no apparent differences were observed in the patient’s clinical condition or medications between the first and second surgeries, and intraoperative ACT measurements were performed using the same reagents and equipment. Nevertheless, a significant difference was observed between the ACT values obtained during the 2 procedures (**Figs. 1** and **2**). This finding suggests that the patient’s antibody levels may have changed over time. Indeed, the antibody titers differed between the first surgery and the serological reassessment performed 12 weeks later. It is therefore possible that the antibody titers also changed between the 12-week reassessment and the second surgery, thereby affecting the ACT measurements. Therefore, even in the same patient undergoing multiple surgeries at different time points, it is essential to generate a new heparin–ACT titration curve before each procedure to establish an accurate, patient-specific target ACT value.

Both revascularization procedures were completed without intraoperative or postoperative complications. Although the preoperative heparin–ACT titration curve provides a practical approach for individualized anticoagulation management, ACT remains an indirect surrogate marker of heparin activity and may not accurately reflect the anticoagulant effect in patients with APS. Therefore, this approach should be regarded as an adjunct to clinical judgment rather than a definitive method for confirming adequate anticoagulation in patients with APS. Further accumulation of cases is required to validate the safety, reliability, and broader applicability of this individualized anticoagulation strategy.

## Conclusion

In this case, intraoperative heparin dosing during lower extremity revascularization in a patient with APS was individualized using a preoperative heparin–ACT titration curve. This approach provided a simple and reliable method for tailoring anticoagulation management and may enhance the safety of peripheral arterial revascularization in patients with APS.

## References

[1] Barbhaiya M, Zuily S, Naden R, et al. The 2023 ACR/EULAR antiphospholipid syndrome classification criteria. Arthritis Rheumatol 2023; 75: 1687–702. 37635643 10.1002/art.42624

[2] Usta A, Yayla ME, Uslu E, et al. The performance of 2023 American College of Rheumatology (ACR)/European Alliance of Associations for Rheumatology (EULAR) antiphospholipid syndrome classification criteria in a real-world rheumatology department. Mediterr J Hematol Infect Dis 2024; 16: e2024074. 39534708 10.4084/MJHID.2024.074 PMC11556423

[3] Massoudy P, Cetin SM, Thielmann M, et al. Antiphospholipid syndrome in cardiac surgery—an underestimated coagulation disorder? Eur J Cardiothorac Surg 2005; 28: 133–7. 15982596 10.1016/j.ejcts.2004.12.062

[4] Koniari I, Siminelakis SN, Baikoussis NG, et al. Antiphospholipid syndrome; its implication in cardiovascular diseases: a review. J Cardiothorac Surg 2010; 5: 101. 21047408 10.1186/1749-8090-5-101 PMC2987921

[5] Miyakis S, Lockshin MD, Atsumi T, et al. International consensus statement on an update of the classification criteria for definite antiphospholipid syndrome (APS). J Thromb Haemost 2006; 4: 295–306. 16420554 10.1111/j.1538-7836.2006.01753.x

[6] Mehta TP, Smythe MA, Mattson JC. Strategies for managing heparin therapy in patients with antiphospholipid antibody syndrome. Pharmacotherapy 2011; 31: 1221–31. 22122183 10.1592/phco.31.12.1221

[7] Gorki H, Malinovski V, Stanbridge RDL. The antiphospholipid syndrome and heart valve surgery. Eur J Cardiothorac Surg 2008; 33: 168–81. 18082413 10.1016/j.ejcts.2007.11.004

[8] Sheu M, Molina Garcia S, Wei W, et al. Efficacy of the Hepcon system in reducing hemorrhagic and thrombotic complications in antiphospholipid syndrome patients undergoing cardiac surgery. Perfusion 2024; 39: 1424–30. 37608561 10.1177/02676591231197990

[9] Seki T, Shingu Y, Sugiki H, et al. Anticoagulation management during cardiopulmonary bypass in patients with antiphospholipid syndrome. J Artif Organs 2018; 21: 363–6. 29541945 10.1007/s10047-018-1032-7

[10] Dornan RI. Acute postoperative biventricular failure associated with antiphospholipid antibody syndrome. Br J Anaesth 2004; 92: 748–54. 15003982 10.1093/bja/aeh116

